# Preoperative Diastolic Dysfunction and Perioperative Risk Factors as Predictors of New Onset Atrial Fibrillation in Patients Undergoing Coronary Artery Bypass Graft Surgery: A Retrospective Cohort Study

**DOI:** 10.21470/1678-9741-2025-0177

**Published:** 2026-05-06

**Authors:** Yan Efrata Sembiring, Lyndon Darwin, Achmad Lefi, Pudji Lestari, Fan Maitri Aldian

**Affiliations:** 1 Department of Thoracic Cardiac and Vascular Surgery, Faculty of Medicine, Universitas Airlangga, Surabaya, Indonesia; 2 Department of Thoracic Cardiac and Vascular Surgery, Dr. Soetomo General Academic Hospital, Surabaya, Indonesia; 3 Department of Cardiology and Vascular Medicine, Faculty of Medicine, Universitas Airlangga, Surabaya, Indonesia; 4 Department of Cardiology and Vascular Medicine, Dr. Soetomo General Academic Hospital, Surabaya, Indonesia; 5 Department of Preventive Medicine and Public Health, Faculty of Medicine, Universitas Airlangga, Surabaya, Indonesia; 6 Medical Program, Faculty of Medicine, Universitas Airlangga, Surabaya, Indonesia

**Keywords:** Cardiopulmonary Bypass Grafting, Diastolic Dysfunction, Postoperative Atrial Fibrillation, Predictor Factor.

## Abstract

**Introduction:**

Despite advancements in technique and the increasing number of coronary artery bypass grafting (CABG) procedures, new-onset postoperative atrial fibrillation (POAF) is one of the most common complications following CABG and remains a major concern. The exact mechanism is unclear, but impaired diastolic function may predispose patients to POAF. Thus, this study aims to evaluate preoperative diastolic dysfunction (DD) and associated factors as predictors of new-onset POAF.

**Methods:**

This retrospective cohort study involved patients undergoing CABG surgery who met the inclusion criteria between January 2018 to August 2022. DD was measured through preoperative transthoracic echocardiogram, while new-onset POAF was assessed through continuous electrocardiogram.

**Results:**

A total of 191 patients who met the inclusion criteria were enrolled in this study. Data-analysis revealed no significant difference in DD between patients with and without POAF (P = 0.72). Multivariate analysis demonstrated left main coronary artery disease (LMCAD) (odds ratio [OR] = 2.51; 95% confidence interval [CI] [1.12 - 5.59]; P = 0.02), in-stent restenosis (ISR) ≥ 70% (OR = 6.34; 95% CI [1.68 - 23.92]; P < 0.01), reduced ejection fraction ≤ 50% (OR = 2.18; 95% CI [0.94 - 5.06]; P = 0.07), and electrolyte imbalance (OR = 2.14; 95% CI [2.91 - 24.75]; P < 0.01) as the independent predictors of new-onset POAF.

**Conclusion:**

DD was not identified as a predictor of new-onset POAF in patients undergoing CABG. The independent predictors identified in this study included male sex, comorbid LMCAD and ISR ≥ 70%, reduced left ventricular ejection fraction ≤ 50%, and postoperative electrolyte imbalance.

## INTRODUCTION

**Table:** 

Abbreviations, Acronyms & Symbols				
AF	= Atrial fibrillation		HFrEF	= Heart failure with reduced ejection fraction
AoX	= Aortic cross-clamping		ICU	= Intensive care unit
AUC	= Area under the curve		ISR	= In-stent restenosis
AUSROC	= Area under the summary receiver operating characteristic curve		LAD LAVI	= Left anterior descending artery = Left atrial volume index
CABG	= Coronary artery bypass grafting		LCx	= Left circumflex artery
CHD	= Coronary heart disease		LMCAD	= Left main coronary artery disease
CI	= Confidence interval		LV	= Left ventricular
CPB	= Cardiopulmonary bypass		LVEF	= Left ventricular ejection fraction
CTO	= Chronic total occlusion		OR	= Odds ratio
DD	= Diastolic dysfunction		POAF	= Postoperative atrial fibrillation
DM	= Diabetes mellitus		RCA	= Right coronary artery
DVD	= Double vessel disease		RCT	= Randomized controlled trial
ECG	= Electrocardiogram		TVD	= Triple vessel disease

Coronary heart disease (CHD) is a leading cause of cardiovascular-related mortality. In 2019 alone, an estimated 17,9 million deaths were attributed to cardiovascular disease^[1]^. Therefore, optimizing the management of CHD patients is crucial, particularly in terms of revascularization. Coronary artery bypass grafting (CABG) surgery is the most commonly performed revascularization method, especially in patients with multi-vessel disease or left main coronary artery disease (LMCAD)^[2]^. Currently, CABG is reported to be performed at a rate of 36,7 per 100.000 population annually^[3]^.

Despite advancements in technique and the increasing number of CABG procedures performed each year, postoperative complications remain a major concern. New-onset postoperative atrial fibrillation (POAF) is one of the most common complications following CABG, with reported incidence ranging from 27% to 41%^[4^,^5]^. POAF is not only associated with increased mortality and morbidity but also contributes to prolonged intensive care unit (ICU) stays, longer hospitalizations, greater healthcare resource utilization, and significantly higher costs. Additionally, POAF increases the risk of stroke, hemodynamic instability, congestive heart failure, neurological events, and renal insufficiency^[6^,^7]^.

Several previous studies have explored patient characteristics that may predict the development of POAF, including older age, male sex, previous history of atrial fibrillation (AF), congestive heart failure, obesity, anemia, impaired renal function, left ventricular (LV) hypertrophy, and left atrial enlargement^[8^-^10]^. Echocardiographic predictors of POAF have also been identified, such as the presence of a patent foramen ovale, increased left atrial area and volume, large left atrial appendage area, low pulmonary vein systolic/diastolic velocity ratio, total atrial conduction time, and decreased left atrial peak systolic strain and early diastolic strain rate^[11^-^13]^.

Diastolic dysfunction (DD), characterized by impaired mechanical function during diastole, is commonly observed in patients undergoing CABG. Several mechanisms may explain the association between DD and AF, including increased atrial afterload, atrial stretch, and atrial wall stress due to dilation^[14]^. While numerous studies have examined predictors of POAF, most have been conducted in developed countries, where patient characteristics, healthcare systems, and intraoperative practices may differ significantly from those in developing regions. To our knowledge, this is the first study to evaluate preoperative DD as a predictor of new-onset POAF in a developing country setting. By incorporating both patient and intraoperative variables, this study aims to fill an important gap in the literature and provide context-specific insights that could enhance preoperative risk assessment and management strategies in resource-limited settings.

## METHODS

### Study Design

This is an observational study with retrospective cohort design. This study involved patients undergoing CABG surgery in Dr. Soetomo General Academic Hospital who met the inclusion criteria between January 2018 to August 2022. Ethical approval was obtained from the Research Ethics Committee of Dr. Soetomo General Hospital (No. 1612/110/4/VIII/2022) on September 13, 2022. The study was conducted in accordance with the principles outlined in the Declaration of Helsinki and Strengthening the Reporting of Observational Studies in Epidemiology (or STROBE) checklist^[15]^.

### Participants

The inclusion criteria for this study were as follows: (1) patients who underwent CABG surgery between January 2018 and August 2022, and (2) adult patients aged over 18 years. Patients with the following characteristics were excluded from the study: (1) history of cardiac arrhythmias prior to surgery, (2) history of valvular heart disease and/or congenital heart defects, (3) history of CABG performed in conjunction with other cardiac surgeries (*e.g.*, valve surgery), and (4) patients with pacemaker. After screening based on the inclusion and exclusion criteria, a total of 191 patients were included in the study. Written informed consent was obtained from all participants for inclusion in the study and for publication purposes. For the surgical procedure, all patients underwent elective on-pump CABG through a median sternotomy. Preoperatively, bisoprolol 2.5 mg once daily was administered as part of routine medical optimization. Upon completion of the procedure, meticulous hemostasis was achieved, and chest drainage was provided using both substernal and intrapleural drains (28 Fr) to ensure adequate evacuation of blood and fluid. The sternum was then closed in the standard manner with steel wires. Patient characteristics collected in this study included age, left ventricular ejection fraction (LVEF), sex, comorbidities (hypertension, diabetes mellitus [DM], anemia, and electrolyte imbalance), and CABG operative characteristics (time of surgery and aortic cross-clamping [AoX] duration). Electrolyte imbalance was identified when one or more serum electrolyte levels were found to be outside the normal reference range, which assessed starting from the postoperative period up to the point of discharge. The normal ranges used in this study were as follows: sodium 135 - 145 mEq/L, potassium 3.5 - 5.0 mEq/L, chloride 96 - 109 mEq/L, magnesium 1.4 - 2.1 mEq/L, and calcium 8.5 - 10.5 mg/dL.

### Assessment of Diastolic Function

All patients scheduled to undergo CABG received a preoperative transthoracic echocardiographic examination. Diastolic function variables were measured using standardized methods. Diastolic parameters were then classified according to the global diastolic function score (grades 0 - 3)^[16]^. Echocardiographic assessments were independently analyzed by two expert echocardiographers in a blinded manner. Any discrepancies in diastolic function grading between the two echocardiographers were resolved through discussion or independent analysis by a third reviewer. Grade I DD indicates impaired myocardial relaxation with normal LV filling pressures. It is characterized by a mitral E/A ratio < 0.75, mitral deceleration time > 240 ms, e′ velocity < 8 cm/s, E/e′ ratio ≤ 8, pulmonary vein systolic flow velocity greater than diastolic flow velocity, pulmonary vein atrial reversal duration shorter than mitral A-wave duration, and left atrial volume index (LAVI) ≥ 34 mL/m^2^. Grade II DD reflects impaired relaxation with mildly to moderately elevated LV filling pressures. It is defined by a mitral E/A ratio between 0.75 and 1.5, with a decrease of ≥ 0.5 during the Valsalva maneuver, mitral deceleration time between 160 and 240 ms, e′ velocity < 8 cm/s, E/e′ ratio of 9 - 12, pulmonary vein systolic flow velocity less than diastolic flow velocity, pulmonary vein atrial reversal duration longer than mitral A-wave duration (≥ 30 ms), and LAVI ≥ 34 mL/m^2^. Grade III DD represents severe DD with a restrictive LV filling pattern. It is characterized by a mitral E/A ratio > 1.5 with a decrease of ≥ 0.5 during the Valsalva maneuver (reversible) or < 0.5 (fixed), mitral deceleration time < 160 ms, e′ velocity < 8 cm/s, E/e′ ratio ≥ 13, pulmonary vein systolic flow velocity less than diastolic flow velocity, pulmonary vein atrial reversal duration longer than mitral A-wave duration (≥ 30 ms), and LAVI ≥ 34 mL/m^2^.

### Assessment of New-Onset Postoperative Atrial Fibrillation

The occurrence of new-onset POAF was monitored through continuous electrocardiogram (ECG) during the intensive care period within the cardiovascular ICU, as well as through daily ECG recordings in the surgical inpatient ward until discharge. A patient was considered to have new-onset POAF if the ECG demonstrated an irregular RR interval (in the absence of atrioventricular conduction abnormalities), absence of distinct repeating P waves, and evidence of irregular atrial activation^[17]^. All POAF diagnoses were confirmed by a cardiologist through interpretation of a 12-lead ECG.

### Statistical Analysis

Categorical data were presented as proportions (%), while continuous data were provided as mean ± standard deviation or median (interquartile range). All data analyses were conducted using the IBM SPSS Statistics, version 27.0 (IBM Corp., Armonk, N.Y., USA). Comparative analyses of all variables between patients with and without new-onset POAF were performed using either the Mann-Whitney U test or the Chi-square test.

Additional analyses were carried out using univariate logistic regression to identify key variables associated with POAF. Subsequently, multivariate logistic regression analysis was conducted to identify independent predictors of POAF. The multivariate models were developed using both forward stepwise and backward stepwise approaches. From the final model of each approach, the beta coefficient (β), odds ratio (OR), 95% confidence interval (CI), and *P*-value were extracted. Model fit was assessed using the Hosmer-Lemeshow goodness-of-fit test, classification table, and the area under the summary receiver operating characteristic (AUSROC) curve. A classification accuracy > 70% was considered indicative of good model fit. An area under the curve (AUC) value < 0.5 was regarded as not acceptable, values > 0.7 indicated an acceptable predictive fit, and an AUC of 1 represented a model with perfect predictive ability. A *P*-value < 0.05 was considered statistically significant for all analyses.

## RESULTS

### Baseline Characteristics of Patients

A total of 191 patients who met the inclusion and exclusion criteria were enrolled in this study. The patients’ characteristics are presented in Table 1. The majority of participants were male (n = 153), with a mean age of 59.67 years, ranging from 36 to 84 years. The mean LVEF was 54.28%, with nearly half of the patients (n = 78; 40.84%) having an LVEF ≤ 50%. A total of 106 patients (55.5%) had a comorbidity of hypertension, while 88 patients (46.1%) had a history of DM.

**Table 1:** Baseline characteristics of the patients according to the occurrence of new-onset POAF.

Characteristic	POAF	Non-POAF	All	*P*-value
	(n = 40)	(n = 151)	(n = 191)	
Age, years	61.58 ± 7.01	59.17 ± 8.82	59.67 ± 8.51	0.18
Age ≥ 60 years	27 (67.5)	83 (54.97)	110 (57.59)	0.15
Sex, n (%)				0.08
Men	36 (90)	117 (77.48)	153 (80.11)	
Women	4 (10)	34 (22.52)	38 (19.89)	
LVEF (%)	56.73 ± 12.24	53.63 ± 12.41	54.28 ± 12.40	0.21
LVEF ≤ 50%, n (%)	13 (32.50)	65 (43.05)	78 (40.84)	0.23
Comorbidity, n (%)				
Hypertension	22 (55)	84 (55.63)	106 (55.5)	0.94
DM	16 (40)	72 (47.68)	88 (46.1)	0.38
Diastolic function, n (%)				0.72
Normal	5 (12.5)	29 (19.21)	34 (17.8)	
Grade 1	31 (77.5)	109 (72.19)	140 (73.3)	
Grade 2	4 (10)	12 (7.95)	16 (8.4)	
Grade 3	0 (0)	1 (0.66)	1 (0.5)	
Vessel disease, n (%)				0.29
DVD	6 (15)	14 (9.27)	20 (10.47)	
TVD	34 (85)	137 (90.73)	171 (89.53)	
LMCAD	26 (65)	68 (45.03)	94 (49.21)	0.03
In-stent restenosis ≥ 70%, n (%)	6 (15)	7 (4.64)	13 (6.81)	0.02
RCA CTO	6 (15)	23 (15.23)	29 (15.18)	0.97
LAD CTO	7 (17.5)	29 (19.21)	36 (18.85)	0.81
LCx CTO	4 (10)	20 (13.25)	24 (12.57)	0.58

Values are presented as mean ± standard deviation or n (%); statistical significance = *P* < 0.05

CTO=chronic total occlusion; DM=diabetes mellitus; DVD=double vessel disease; LAD=left anterior descending artery; LCx=left circumflex artery; LMCAD=left main coronary artery disease; LVEF=left ventricular ejection fraction; POAF=postoperative atrial fibrillation; RCA=right coronary artery; TVD=triple vessel disease

Among all participants, almost all were diagnosed with triple vessel disease (n = 171). Several concomitant conditions were also observed, including LMCAD (n = 94), in-stent restenosis (ISR) ≥ 70% (n = 13), chronic total occlusion (CTO) of the right coronary artery (n = 29), CTO of the left anterior descending artery (n = 36), and CTO of the left circumflex artery (n = 24). Diastolic function assessment using echocardiography indicated that the majority of patients exhibited grade 1 DD (n = 140), while 16 and one patients were found to have grade 2 and grade 3 DD, respectively. Additionally, 34 patients were identified as having normal diastolic function.

### Incidence of New-Onset Postoperative Atrial Fibrillation, Intraoperative Parameters, and Postoperative Findings

The overall incidence rate of new-onset POAF was 20.94% (n = 40), with most cases occurring on the second postoperative day, ranging from the first to the fifth day following CABG. Sinus rhythm was successfully restored in 38 patients (95%) prior to hospital discharge (refer to Table 2). Based on the distribution of new-onset POAF cases presented in Table 1, there was no significant difference in DD between patients with and without POAF (*P* = 0.72). Overall, patients with LMCAD (*P* = 0.03) and ISR ≥ 70% (*P* = 0.02) were more likely to develop POAF compared to those without these conditions.

**Table 2:** Occurrence and clinical development of new-onset POAF.

Variable	All patients (n = 191)
New-onset POAF, n (%)	40 (20.94)
Onset, day	2.1 ± 0.84
Sinus conversion, n (%)	38 (95)
POAF=postoperative atrial fibrillation	

Data related to intraoperative characteristics and postoperative complications are presented in Table 3. No significant difference was observed in the mean cardiopulmonary bypass time between patients who developed POAF (120.4 minutes) and those who did not (118.98 minutes; *P* = 0.94). A similar finding was observed for the AoX time, with a mean duration of 74.83 minutes across all patients (*P* = 0.50). Regarding postoperative complications, patients who developed anemia (27.5%) were significantly more likely to experience POAF compared to non-anemic patients (8.61%; *P* = 0.001). In addition, electrolyte disturbances were associated with a markedly increased risk of POAF (*P* < 0.001). On the other hand, no significant difference in hospital mortality was found between patients with and without POAF (*P* = 0.19).

**Table 3:** Intraoperative characteristics and postoperative complications regarding CABG based on the occurrence of new-onset POAF.

Characteristic	POAF	Non-POAF	All	*P*-value
	(n = 40)	(n = 151)	(n = 191)	
Intraoperative data				
CPB time, minutes	120.4 ± 33.39	118.98 ± 37.38	119.28 ± 36.51	0.94
AoX time, minutes	73.53 ± 25.04	75.17 ± 22.5	74.83 ± 22.99	0.50
Postoperative complication, n (%)				
Anemia	11 (27.5)	13 (8.61)	24 (12.6)	0.001
Electrolyte imbalance	11 (27.5)	8 (5.30)	19 (9.9)	< 0.001
Mortality	4 (10)	7 (4.64)	11 (5.8)	0.195

Values are presented as mean ± standard deviation or n (%); statistical significance = *P* < 0.05

AoX=aortic cross-clamping; CPB=cardiopulmonary bypass; POAF=postoperative atrial fibrillation

### Predictive Modelling of New-Onset Postoperative Atrial Fibrillation using Univariate and Multivariate Logistic Regression

The results of the univariate and multivariate logistic regression analyses on patient characteristics, comorbidities, diastolic function, intraoperative variables, and postoperative complications are presented in Table 4 and Table 5, respectively. Variations in DD grading were not identified as significant predictors of POAF occurrence, including DD grade 1 (OR = 0.61; 95% CI [0.22 - 1.69]; *P* = 0.34), DD grade 2 (OR = 0.52; 95% CI [0.12 - 2.26]; *P* = 0.38), and DD grade 3 (OR = 0.27; 95% CI [0.01 - 5.62]; *P* = 0.99). Regarding coexisting conditions, patients with LMCAD (OR = 0.44; 95% CI [0.21 - 0.91]; *P* = 0.02) and ISR ≥ 70% (OR = 3.63; 95% CI [1.14 - 11.49]; *P* = 0.02) were found to be significant predictors of POAF. Additionally, the univariate analysis revealed other predictive factors related to postoperative complications, such as anemia (OR = 0.25; 95% CI [0.10 - 0.61]; *P* < 0.01) and electrolyte imbalance (OR = 6.78; 95% CI [2.51 - 18.32]; *P* < 0.01).

**Table 4:** Univariate analysis predictors of new-onset POAF.

Covariate	OR	95% CI	*P*-value
Age, years	0.97	0.93 - 1.01	0.11
Age ≥ 60 years	0.59	0.28 - 1.23	0.16
Sex	2.61	0.87 - 7.87	0.09
LVEF	0.98	0.95 - 1.01	0.98
LVEF ≤ 50%	1.57	0.75 - 3.27	0.23
Hypertension	0.97	0.48 - 1.97	0.94
DM	1.37	0.67 - 2.77	0.38
Diastolic Grade 1	0.61	0.22 - 1.69	0.34
Diastolic Grade 2	0.52	0.12 - 2.26	0.38
Diastolic Grade 3	0,27	0.01 - 5.62	0.99
DVD	1.73	0.62 - 4.82	0.29
TVD	0.58	0.21 - 1.62	0.29
LMCAD	0.44	0.21 - 0.91	0.02
In-stent restenosis	3.63	1.14 - 11.49	0.02
RCA CTO	1.02	0.38 - 2.69	0.97
LAD CTO	0.89	0.36 - 2.22	0.81
LCx CTO	1.37	0.44 - 4.27	0.58
CPB time	0.99	0.99 - 1.01	0.83
AoX time	1.00	0.98 - 1.02	0.69
Anemia	0.25	0.10 - 0.61	< 0.01
Electrolyte imbalance	6.78	2.51 - 18.32	< 0.01
Mortality	2.29	0.63 - 8.23	0.21

Statistical significance = *P* < 0.05

AoX=aortic cross-clamping; CI=confidence interval; CPB=cardiopulmonary bypass; CTO=chronic total occlusion; DM=diabetes mellitus; DVD=double vessel disease; LAD=left anterior descending artery; LCx=left circumflex artery; LMCAD=left main coronary artery disease; LVEF=left ventricular ejection fraction; OR=odds ratio; POAF=postoperative atrial fibrillation; RCA=right coronary artery; TVD=triple vessel disease

**Table 5:** Multivariate logistic regression analysis on predictors of new-onset POAF.

Covariate	β	OR	95% CI	*P*-value
Male	1.36	3.89	1.13 - 13.32	0.03
LMCAD	0.91	2.51	1.12 - 5.59	0.02
In-stent restenosis ≥ 70%	1.85	6.34	1.68 - 23.92	< 0.01
LVEF ≤ 50%	0.78	2.18	0.94 - 5.06	0.07
Electrolyte imbalance	2.14	8.49	2.91 - 24.75	< 0.01

Statistical significance = *P* < 0.05; R^2^ = 0.245, Hosmer-Lemeshow test significancy = 0.756, correctly classified = 83.2%, area under the summary receiver operating characteristic curve = 0.75

CI=confidence interval; LMCAD=left main coronary artery disease; LVEF=left ventricular ejection fraction; OR=odds ratio

To assess the predictive strength of factors identified in the univariate analysis, a multivariate logistic regression analysis was conducted including all covariates (Table 5). The multivariate model showed that male patients had a 3,89-fold higher risk of developing POAF compared to female patients (*P* = 0.03). Other independent predictors of new-onset POAF included LMCAD (OR = 2.51; 95% CI [1.12 - 5.59]; *P* = 0.02), ISR ≥ 70% (OR = 6.34; 95% CI [1.68 - 23.92]; *P* < 0.01), and electrolyte imbalance (OR = 2.14; 95% CI [2.91 - 24.75]; *P* < 0.01). Furthermore, the model also indicated that LVEF ≤ 50% (OR = 2.18; 95% CI [0.94 - 5.06]; *P* = 0.07) may serve as a potential predictor, although it was not statistically significant. The reversal of OR for LMCAD from 0.44 in the univariate analysis to 2.51 in the multivariate model, likely reflects the influence of confounding factors that were accounted for the multivariate analysis. The AUSROC curve for the multivariate prediction model was 0.75, indicating strong predictive power, with 83.2% of patients correctly classified by the model.

## DISCUSSION

This study found the prevalence of new-onset POAF among patients undergoing CABG to be 20.9%. POAF is a common complication of CABG, occurring in approximately 5% to 40% of patients during the early postoperative period. Typically, POAF manifests between the second and fourth day after surgery, with the peak incidence occurring on postoperative day two^[5^,^18]^. This finding aligns with the results of the present study, in which the average onset of POAF occurred on the second postoperative day. During this time frame, patients are generally weaned off anesthetic medications, extubated, and provided with postoperative care aimed at preventing complications. Because this period is characterized by intensive monitoring of ECG changes and vital signs, it represents an optimal window for detecting POAF and initiating early management if AF is identified^[19]^. Current evidence suggests that new-onset POAF in post-CABG patients may be triggered by the formation of an arrhythmogenic atrial substrate that initiates arrhythmias^[20]^. POAF may also be stimulated by various factors that disrupt normal cardiac electrical conduction, highlighting the importance of identifying predictors of POAF to minimize risk and enable timely intervention.

The occurrence of POAF in patients undergoing CABG has important clinical implications. Consistent with previous studies, POAF was associated with prolonged ICU stay and overall hospitalization, likely reflecting the need for rhythm monitoring, pharmacologic intervention, and management of hemodynamic instability^[6^,^21]^. The development of POAF is not only associated with prolonged ICU stay and length of hospitalization but also with an increased risk of postoperative complications. Patients with POAF are more likely to experience hemodynamic instability, perioperative myocardial ischemia, stroke, and renal dysfunction, which collectively contribute to worse short-term outcomes^[7^,^22]^. In addition, POAF has been linked to higher rates of infection and bleeding due to the need for anticoagulation or extended invasive monitoring^[23]^. These findings emphasize that POAF represents more than a transient arrhythmia; it is an important postoperative complication that adversely affects recovery and may increase morbidity following CABG.

In this study, the proportion of DD among patients undergoing CABG was 82.2%. The study found no significant association between DD and new-onset POAF in CABG patients. This finding contrasts with that of Melduni et al. (2011), who reported that the incidence of POAF increases exponentially with greater severity of DD^[10]^. Similarly, Ashes et al. (2014) found that new or worsened DD was an independent predictor of POAF^[9]^. However, a randomized controlled trial (RCT) involving patients undergoing cardiac surgery also reported that DD was not a predictor of POAF in multivariate analysis^[24]^. The discrepancy in findings among studies may be attributed to differences in the criteria used to assess diastolic function. This study employed the criteria proposed by Nagueh et al. (2009), whereas other studies utilized criteria from earlier research that reported significant echocardiographic parameters^[25^-^28]^. A key difference lies in the thresholds for the E/A ratio and mitral deceleration time. Additionally, in patients with hypertrophic cardiomyopathy, assessing DD can be challenging due to significant variations in mitral inflow patterns and individual differences in ventricular compliance and relaxation^[24]^. Moreover, the assessment of diastolic function is subject to inter-observer variability, making DD difficult to include as a reliable component of predictive models for postoperative AF.

Independent predictors of new-onset POAF identified in this study include male sex, comorbid LMCAD and ISR ≥ 70%, LVEF ≤ 50%, and postoperative complications such as electrolyte imbalance. Previous evidence has also identified independent predictors of POAF such as advanced age^[9^,^10^,^24^,^29^,^30]^, elevated body mass index^[10^,^29^,^31]^, DD^[9^,^10]^, increased left atrial volume^[24]^, reduced LVEF^[29^,^30]^, and elevated initial intraoperative pulmonary artery pressure^[24]^. An RCT involving patients with LMCAD reported that the incidence of new-onset POAF was higher in patients undergoing CABG (18%) compared to those undergoing percutaneous coronary intervention (0.1%)^[29]^. Other studies have noted that POAF is increasingly prevalent in patients with heart failure with reduced ejection fraction (HFrEF), likely due to shared pathophysiological and mechanistic features between AF and HFrEF^[32^,^33]^.

Electrolytes such as potassium, magnesium, calcium, and phosphate are essential for cellular metabolism and membrane potential regulation. Patients undergoing cardiac surgery with extracorporeal circulation are prone to electrolyte imbalances due to hypothermia-induced diuresis and intracellular shifts, despite the use of potassiumand magnesium-rich cardioplegia and intraoperative potassium supplementation^[34]^. Electrolyte imbalances can lead to abnormal atrial automaticity, self-sustaining action potentials, or re-entry circuits^[35]^. According to the literature, potassium is a key electrolyte influencing the incidence of POAF. A study by Howitt et al. (2020) reported that maintaining serum potassium concentrations at ≥ 4.5 mmol/L post-cardiac surgery can reduce the incidence of POAF^[36]^.

Identifying risk factors for postoperative AF in CABG patients is essential for implementing effective AF prevention strategies and enabling timely management. Although various risk factors have been reported in previous studies, the present study contributes additional independent predictors that can optimize the management of patients with preoperative DD undergoing CABG.

### Strength and Limitations

The primary strength of this study lies in its relatively adequate sample size, which is sufficient to represent the target population. It also includes patients with diverse characteristics, encompassing a wide age range, and evaluates several additional risk factors that have not been previously established as predictors in earlier studies, including ISR ≥ 70%, LMCAD comorbidity, anemia, and postoperative electrolyte imbalance. Although numerous similar studies have been published to date, to the best of the authors’ knowledge, this is the first study conducted in a developing country where patient characteristics and intraoperative data may differ significantly from those in developed countries. These findings have important clinical implications for the early identification and management of patients at risk of developing POAF following CABG surgery. Recognizing preoperative DD and specific perioperative risk factors as predictors of POAF may help guide perioperative monitoring strategies and target preventive interventions. By stratifying patients based on these risk factors, clinicians may implement more personalized approaches, such as closer hemodynamic surveillance, optimization of fluid balance, and early consideration of pharmacologic prophylaxis. Ultimately, incorporating these predictors into routine perioperative risk assessment may improve patient outcomes and reduce the incidence of POAF-related complications.

Nevertheless, despite efforts to produce a high-quality study, several limitations must be acknowledged. First, this study assessed DD only in a global context and did not analyze the relationship between individual echocardiographic parameters of DD and the incidence of POAF following CABG, such as LV mass and mass index, left atrial volume, E and A wave velocities, E/A ratio, and deceleration time, as performed in previous literature^[10^,^24]^. Second, variability in the diagnostic criteria for DD across studies may lead to heterogeneity in findings. Third, detailed information regarding beta-blocker use, type of surgical drains, drain techniques, and other surgical characteristics was not consistently recorded in the medical records and therefore could not be reliably analyzed. Consequently, the potential influence of these variables on the development of POAF could not be assessed in this study. Lastly, the study did not include long-term follow-up, thus limiting the ability to track the occurrence of POAF over an extended period.

## CONCLUSION

DD was not identified as a predictor of new-onset POAF in patients undergoing CABG. The independent predictors identified in this study included male sex, comorbid LMCAD and ISR ≥ 70%, reduced LVEF ≤ 50%, and postoperative electrolyte imbalance. Further studies are warranted to confirm these findings in larger populations. Given the variability in results across similar studies, the authors recommend conducting a comprehensive review that synthesizes findings from all available primary studies to better determine interrelated predictive factors.

## Data Availability

The authors declare that the data supporting the findings of this study are available within the article and that data sharing is not applicable to this article.

## Artificial Intelligence Usage

The authors declare that no artificial intelligence tool was used in the preparation of this article.
